# Condition-dependent generation of aquaporin-4 antibodies from circulating B cells in neuromyelitis optica

**DOI:** 10.1093/brain/awy010

**Published:** 2018-02-13

**Authors:** Robert Wilson, Mateusz Makuch, Anne-Kathrin Kienzler, James Varley, Jennifer Taylor, Mark Woodhall, Jacqueline Palace, M Isabel Leite, Patrick Waters, Sarosh R Irani

**Affiliations:** 1 Oxford Autoimmune Neurology Group, Nuffield Department of Clinical Neurosciences, University of Oxford, Oxford, UK; 2 Oxford University Hospitals, John Radcliffe Hospital, Oxford, OX3 9DS, UK

**Keywords:** neuromyelitis optica, neuroimmunology, neuroinflammation, demyelination, antibodies

## Abstract

Autoantibodies to aquaporin-4 (AQP4) are pathogenic in neuromyelitis optica spectrum disorder (NMOSD). However, it is not known which B cells are the major contributors to circulating AQP4 antibodies nor which conditions promote their generation. Our experiments showed CD19^+^CD27^++^CD38^++^ circulating *ex vivo* antibody-secreting cells did not produce AQP4 antibodies under several culture conditions. To question whether other cells in circulation were capable of AQP4 antibody production, B cells were differentiated into antibody-secreting cells *in vitro*. Unfractionated peripheral blood mononuclear cells, isolated from 12 patients with NMOSD and a wide range of serum AQP4 antibody levels (91–26 610 units), were cultured with factors that mimicked established associations of NMOSD including T cell help, concurrent infections and cytokines reported to be elevated in NMOSD. Overall, the *in vitro* generation of CD19^+^CD27^++^CD38^++^ cells across several culture conditions correlated closely with the total IgG secreted (*P < *0.0001, r = 0.71), but not the amount of AQP4 antibody. AQP4 antibody production was enhanced by CD40-ligand (*P = *0.005), and by interleukin-2 plus toll-like receptor stimulation versus interleukin-21-predominant conditions (*P < *0.0001), and did not require antigen. Across NMOSD patients, this *in vitro* generation of AQP4 antibodies correlated well with serum AQP4 antibody levels (*P = *0.0023, r = 0.81). To understand how early within B cell lineages this AQP4 specificity was generated, purified B cell subsets were activated under these optimized conditions. Naïve pre-germinal centre B cells (CD19^+^CD27^−^IgD^+^) differentiated to secrete AQP4 antibodies as frequently as post-germinal centre cells (CD19^+^CD27^+^). Taken together, these human cell-culture experiments demonstrate that preformed B cells, rather than *ex vivo* circulating antibody-secreting cells, possess AQP4 reactivity. Their differentiation and AQP4 antibody secretion is preferentially driven by select cytokines and these cells may make the dominant contribution to serum AQP4 antibodies. Furthermore, as AQP4-specific B cells can derive from likely autoreactive naïve populations an early, pre-germinal centre loss of immunological tolerance appears present in some patients with NMOSD. This study has implications for understanding mechanisms of disease perpetuation and for rational choice of immunotherapies in NMOSD. Furthermore, the *in vitro* model presents an opportunity to apply condition-specific approaches to patients with NMOSD and may be a paradigm to study other antibody-mediated diseases.

## Teaser

Aquaporin-4 antibodies are pathogenic in neuromyelitis optica spectrum disorders (NMOSD). Wilson *et al.* show that circulating memory and naïve B cells from patients can differentiate to produce aquaporin-4 antibodies under conditions chosen to mimic aspects of NMOSD. This platform should direct rationale immunotherapy in NMOSD and other antibody-mediated conditions.

## Introduction

Immunoglobulin G (IgG) autoantibodies against the extracellular domain of aquaporin-4 (AQP4) are likely pathogenic mediators of neuromyelitis optica spectrum disorders (NMOSD) (Lennon *et al.*, 2005; Wingerchuk *et al.*, 2015). Antibody-mediated fixation of complement and internalization of AQP4, alongside associated proteins, are established as major mechanisms of disease causation (Hinson *et al.*, 2012). However, the cells that generate AQP4 antibodies and conditions that may promote this have received relatively little attention (Bennett *et al.*, 2009; Chihara *et al.*, 2011).

Classical models of plasma cell generation (Supplementary Fig. 1) describe antibodies being produced by antibody-secreting cells (ASCs), which include proliferative circulating plasmablasts and non-proliferative tissue-resident short- and long-lived plasma cells. The precursors to ASCs initially emerge from the bone marrow as antigen-inexperienced naïve B cells. Subsequently, typically in a germinal centre, these naïve B cells develop memory for an antigen, acquire surface CD27, and either switch their immunoglobulin class to express surface IgG (switched memory B cells) or remain unswitched with surface IgD/IgM expression (unswitched memory B cells) (Dalakas, 2008; LeBien and Tedder, 2008). To gain memory for antigen, the germinal centre B cells traditionally require antigen-specific T cell help. Indeed, AQP4-specific T cells are described in patients with NMOSD (Varrin-Doyer *et al.*, 2012). Subsequently, antigen-experienced memory B cells may persist or differentiate into the earliest circulating ASCs, also termed plasmablasts. Plasmablasts can mature into short- or long-lived plasma cells. The long-lived plasma cells typically migrate to bone marrow niches and may secrete constant levels of IgGs for many years (Hoyer *et al.*, 2008; Hiepe *et al.*, 2011; Halliley *et al.*, 2015). Importantly, cell-surface expressed markers vary across these B cell populations (Supplementary Fig. 1). Hence, identification of the B cell subsets that generate AQP4-IgG may help direct more targeted immunotherapies.

Indeed, AQP4-specific plasma cells have been identified in the CSF of patients with NMOSD (Bennett *et al.*, 2009). However, soluble AQP4-IgGs are observed at ∼1000-fold greater levels in blood than in CSF and may be undetectable in CSF (Majed *et al.*, 2016), suggesting the original generation of AQP4-IgGs is in the periphery. Furthermore, NMOSD patients with AQP4-IgGs have elevated serum levels of cytokines involved in T and B cell function (for example IL-2) and in the maintenance of ASCs, including IL-6, APRIL and BAFF (Linhares *et al.*, 2013; Wang *et al.*, 2013; Jourdan *et al.*, 2014; Kothur *et al.*, 2016). These collective observations suggest that the cytokine milieu, including T cell help, may promote generation of AQP4-IgGs from peripheral B cells.

With greater relevance to peripheral B cells, it has been reported that circulating ASCs (plasmablasts; CD19^+^CD27^++^CD38^++^) are elevated in patients with NMOSD, and these ASCs secrete AQP4-IgGs in culture with IL-6 (Chihara *et al.*, 2011). Yet, several clinical observations suggest this may not be the predominant mechanism of AQP4-IgG production. Circulating ASCs typically have a short life-span, and therefore may not show the longevity required to sustain the observed AQP4-IgG production over several years, sometimes decades (Leite *et al.*, 2012). Furthermore, circulating ASCs often downregulate CD20 and are, therefore, relatively insensitive to rituximab, an effective treatment for NMOSD (Damato *et al.*, 2016). By contrast, long-lived plasma cells are a non-replicative population that do not express CD20; therefore, medications such as azathioprine, mycophenolate mofetil or methotrexate, and therapeutic antibodies against CD20, should not target long-lived plasma cells. Yet, these drugs have proven observational efficacy in NMOSD (Jacob *et al.*, 2009; Costanzi *et al.*, 2011; Damato *et al.*, 2016). By contrast, the long-lived plasma cells may be targeted by bone marrow ablative therapy, which has successfully reduced serum AQP4-antibody levels in a few patients (Greco *et al.*, 2014). Therefore, available observations have divergent implications for the relative contributions of B lineage cells to AQP4-IgG production.

Taken together, identification of the cells that produce AQP4-antibodies has clear therapeutic implications for patient- and cell-specific biologics in NMOSD. Furthermore, the position of AQP4-specific cells within the B cell lineage has implications for where B cell tolerance may be disrupted in NMOSD (Bennett *et al.*, 2015; Melchers, 2015). Hence, we established human cell cultures to study the *in vitro* capacity of peripheral B cells to differentiate and produce AQP4-IgGs, and explored conditions that promoted the generation of these autoreactive antibodies. Moreover, we aimed to appreciate the contribution of these cells to serum AQP4-IgG levels and understand B cell tolerance checkpoints in this condition.

## Materials and methods

### Participants

Twelve patients with NMOSD from the Oxford specialist clinic were selected with widely-varying serum AQP4-IgG levels [Table 1, 91–26 610 ΔMFI (median fluorescence intensity) units] and durations from disease onset. Clinical datasets, including patient demographics, presenting features, medications and relapses timings, were extracted from case notes. Blood was obtained from these 12 patients and from 12 sex- and age-matched (±5 years) healthy control subjects. Full informed consent was obtained and the work was performed under Research ethics committee approvals 16/YH/0013 and 16/SC/0224.

**Table 1:** Clinical characteristics of patients with NMOSD

Patient number	Age at sampling	Sex	Ethnicity	Disease localization	Time since first event (days)	Time since last relapse (days)	Duration of IT (days)	Current IT (daily dose)	Serum AQP4-IgG level (ΔMFI)
1	41	F	Caucasian	TM	1741	664	1199	Pred 15 mg	26 610
2	67	F	Caucasian	TM	1224	1141	1218	AZA 125 mg	15 267
3	36	F	Asian	ON and TM	7653	72	1442	Pred 20 mg; MMF 3 g	6126
4	50	F	African	TM	433	433	413	Pred 25 mg; MMF 1.5 g	3080
5	44	M	Caucasian	TM	2364	93	2344	Pred 10 mg; MMF 2.5 g	1863
6	55	F	Caucasian	ON	1728	1671	1708	MMF 2.5 g	1742
7	67	F	Caucasian	TM	3411	1798	1788	Pred 10 mg; MMF 2 g	1350
8	75	F	Caucasian	TM	688	688	668	Pred 10 mg; MMF 2 g	422
9	52	F	Caribbean	ON and TM	1125	667	1120	Pred 15 mg; MMF 2 g	209
10	18	F	African	Brain, TM	12	12	12	Pred 40 mg	91
11	57	F	Caucasian	TM	8589	2958	3108	RTX	402
12	37	F	Caribbean	Brain, ON and TM	3609	47	3243	Pred 20 mg; RTX	235

Serum AQP4-IgG levels determined by flow cytometry as described in Fig. 3 (ΔMFI, as defined in Methods). AZA = azathioprine; IT = immunotherapy; MMF = mycophenolate mofetil; ON = optic neuritis; Pred = prednisolone; TM = transverse myelitis; RTX = rituximab. Only Patients 11 and 12 ever received rituximab. Lymphocyte subsets are presented in Supplementary Table 1.

### Flow cytometry and cell sorting

From patients and controls, peripheral blood mononuclear cells (PBMCs) were isolated on a Ficoll density gradient. Six of 12 unfractionated PBMC samples were studied fresh, and the other half frozen on liquid nitrogen, as detailed in the Supplementary material. To determine *ex vivo* surface B cell phenotypes (Supplementary Fig. 1), PBMCs were labelled at 4°C with antibodies against CD3 (clone UCHT1, Pacific Blue, BioLegend), CD14 (clone HCD14, Pacific Blue, BioLegend), CD19 (clone SJ25C1, APC-Cy7, BD Biosciences), CD27 (clone O323, BV605, BioLegend), CD20 (clone 2H7, FITC, BD Biosciences), IgD (clone IA6-2, PE-CF594, BD Biosciences), CD38 (clone HB7, PE-Cy7, BD Biosciences) and CD138 (clone B-B4, PE, Miltenyi Biotec). Subsequently, cells were washed in PBS/0.1% bovine serum albumin, and DAPI was added prior to analysis with a BD LSRII flow cytometer. For cell-sorting experiments, a FACS Aria III was used to purify selected B cell populations, including ASCs, from fresh PBMC samples. For determination of all cell phenotypes, populations were gated as CD3^−^CD14^−^DAPI^−^ prior to B cell (CD19) analyses. Throughout, FlowJo v10.1r5 was used for analysis.

### Cell culture

For cell culture experiments, 2 × 10^5^ unfractionated PBMCs per well were plated in RPMI (supplemented with 5% IgG-depleted foetal calf serum, penicillin-streptomycin, l-glutamine, IgG-depleted transferrin and 2-mercaptoethanol) and incubated in flat-bottomed 96-well plates with a variety of cytokines and stimulants namely, R848 (2.5 μg/ml Enzo Life Sciences), soluble CD40-ligand (sCD40L; 50 ng/ml, R&D Systems), interleukin-2 (IL-2; 50 ng/ml PeproTech), interleukin-1β (IL-1β; 1 ng/ml PeproTech), interleukin-21 (IL-21; 50 ng/ml PeproTech), interleukin-6 (IL-6; 10 ng/ml R&D Systems), tumour necrosis factor-α (TNFα; 1 ng/ml PeproTech), B cell activating factor (BAFF; 200 ng/ml R&D Systems), and a proliferation inducing ligand (APRIL; 300 ng/ml R&D Systems). To permit cross-linking, some experiments involved co-cultures with membrane bound CD40L (mCD40L)-expressing 3T3 cells, post-irradiation at 70 Gy. After 6 days *in vitro*, cells were analysed by flow cytometry as described. As preliminary data suggested autologous T cells efficiently activated fractionated B cell subsets, CD4^+^ T cells (purified with Dynabeads® Untouched Human CD4^+^ T cell kit, Invitrogen) were mitomycin C-treated (Sigma Aldrich) before incubation on CD3 antibody-coated plates (BD Biosciences) and co-cultured with B cell subsets (purity >99%). For cultures with fresh purified ASCs, sorted cells were incubated with IL-6 in the presence or absence of M2-10B4 bone marrow stromal cells (gift from Prof. R. Tooze).

### Quantitative PCR

To further characterize B cell populations *in vitro*, RNA was extracted from sorted B cells at Days 0 and 7, (ReliaPrep^™^ RNA Cell Miniprep System, Promega) and cDNA synthesized with SuperScript^®^ III reverse transcriptase (Invitrogen) and a Random Primer Mix (New England Biolabs). Quantitative PCR was performed with the TaqMan^®^ Gene Expression Master Mix (Applied Biosystems) using primers against *PRDM1* (gene encoding BLIMP1), *XBP1*, *IRF4*, *PAX5* and *BCL6*. Messenger RNA levels were standardized to the reference Human *RPLPO* Endogenous Control (Applied Biosystems). Day 7 results were expressed as a fold-change over Day 0. The PCR protocol and primers have been described in more detail previously (Kienzler *et al.*, 2013).

### Determination of IgG and AQP4-IgGs

After 13 days *in vitro*, from parallel cell cultures, 5 µl of cell supernatants were tested for total IgG by ELISA (Bethyl Laboratories kit). Also, 50 µl was used to determine presence of AQP4-IgGs using a live immunofluorescent cell-based assay validated previously (Waters *et al.*, 2016). First, visual assessment of the cell-based assay was performed blind to the culture condition. Subsequently, at non-saturating quantities, cell-based assay-positive supernatants (10–50 µl) were quantified using flow cytometry against 10^4^ live HEK cells transfected with a plasmid co-expressing M23-AQP4 and dsRed. Briefly, after washing, bound IgG was detected with an anti-human IgG Alexa Fluor^®^ 488-conjugated secondary antibody (1:500, Jackson Immunoresearch). AQP4-IgGs were quantified as the MFI of dsRed-expressing cells minus the MFI of cells that did not express dsRed (ΔMFI). For serum AQP4-IgG quantification, 0.1–5 µl was used with 2 × 10^5^ AQP4-transfected live HEK cells. Tetanus toxoid-IgGs were determined by ELISA (IU/ml; Binding site kit MK010).

Throughout, GraphPad Prism 7 was used for statistical analyses and data presentation.

## Results

### Patient characteristics

As described in Table 1, the 12 patients showed features consistent with AQP4-antibody-related diseases and fulfilled criteria for NMOSD (Wingerchuk *et al.*, 2015). Eleven of 12 (92%) were female, and patients had a wide age distribution at disease onset (median 51 years, range 18–75), with a high non-Caucasian representation (7/12, 58%). PBMCs were sampled at a median of 1174 days (range 12–8589) after disease onset at which time patients were taking prednisolone plus mycophenolate mofetil (*n = *6), prednisolone only (*n = *2), azathioprine or mycophenolate mofetil alone (*n = *2) or rituximab (*n = *2, administered 12 months and 6 months prior to sampling). Typically, patients had persistent serum AQP4-IgG levels despite several years of immunotherapy, as is well-recognized in NMOSD (Jarius *et al.*, 2008).

#### B cell culture conditions and the *in vitro* generation of antibody-secreting cells

**Figure 1:**
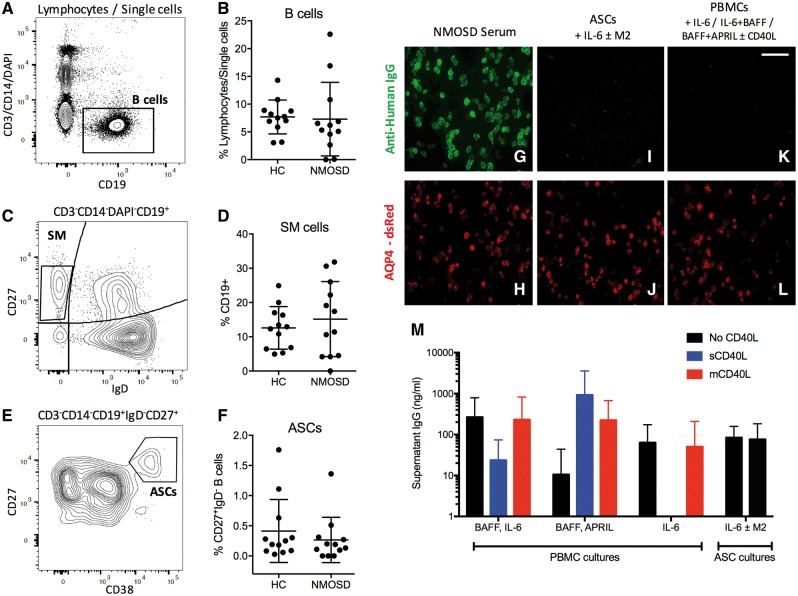
**B cell flow cytometry from patients with AQP4-IgG positive NMOSD and healthy controls.** *Ex vivo* PBMCs from patients and healthy control subjects (HCs) gated as single CD3^−^CD14^−^DAPI^−^CD19^+^ B lymphocytes (**A** and **B**), CD27^+^IgD^−^ switched memory cells from the B cell gate (SM; **C** and **D**) and CD27^++^CD38^++^ (ASCs; **E** and **F**) from the CD27^+^IgD^−^ gate. As expected, NMOSD patient serum IgG bound the surface of AQP4 and dsRed co-expressing cells (**G** and **H**, magnification ×400). After 13 days in culture, AQP4-IgG was not detected from sorted patient ASCs [cultured with IL-6 and/or the bone marrow stromal cell line, M2-10B4 (M2); **I** and **J**] or from PBMCs cultured under ASC-maintenance conditions (IL-6; BAFF with IL-6; BAFF with APRIL, all ± CD40L; **K** and **L**). Total IgG determination from PBMC and ASC cultures from 12 patients was comparable to literature values (**M**). Scale bar = 100 µm.

*Ex vivo* B cell subsets in these 12 patients and matched healthy controls were compared by flow cytometry, and showed no differences between proportions of total B cells (CD19^+^, Fig. 1A and B), and B cell subsets including switched memory B cells (CD19^+^IgD^−^CD27^+^, Fig. 1C and D) and ASCs (CD19^+^IgD^−^CD27^++^CD38^++^, Fig. 1E, F and Supplementary Table 1). Medications administered to patients did not appear to alter B cell subsets (Supplementary Fig. 2).

From the six patients with the highest serum AQP4-IgG levels, 6000–12 500 purified ASCs per well (gating in Fig. 1E), similar numbers to a previous study (Chihara *et al.*, 2011), did not produce AQP4-IgGs after culture with IL-6, or with IL-6 plus the ASC-cell supporting M2-10B4 stromal bone-marrow cell line (Fig. 1G–J). Moreover, no AQP4-IgGs were detected in supernatants from unfractionated PBMC cultures upon supplementation with ASC-maintaining factors including IL-6, BAFF and APRIL (Fig. 1K and L). The absence of AQP4-IgGs from both isolated ASC and unfractionated PBMC culture supernatants was observed despite detection of total IgGs, at levels comparable to other studies (Fig. 1M) (Chihara *et al.*, 2011; Lanzavecchia and Corti, 2014). This absence led us to question whether other peripheral B cells showed the capacity to produce AQP4-IgGs.

**Figure 2:**
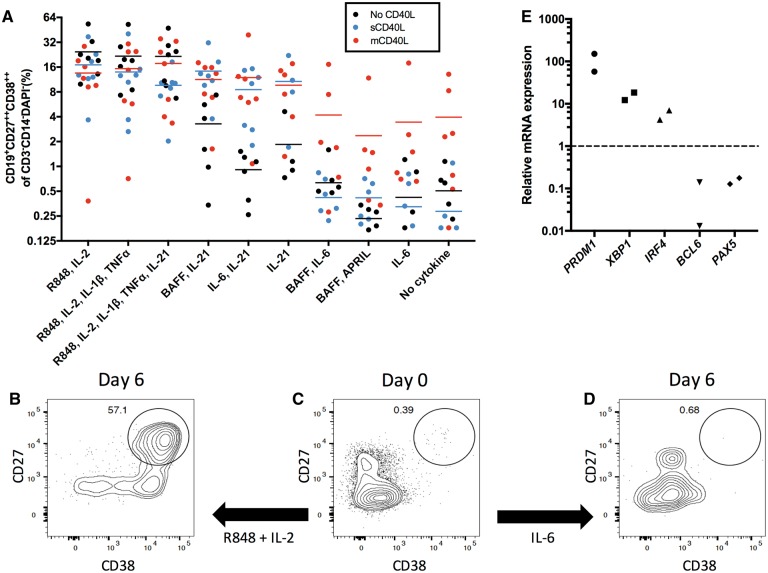
**The generation of ASCs in culture.** The percentage of ASCs (gated as the CD19^+^CD27^++^CD38^++^ of the CD3^−^CD14^−^DAPI^−^ cells) generated after 6 days in culture under a range of conditions alone (black) or with soluble CD40L (sCD40L, blue) or membrane-bound CD40L (mCD40L, red). Results from seven patients (**A**). Illustrative flow cytometry plots (**B**–**D**) show the Day 0 *ex vivo* CD27^++^CD38^++^ cell population (**C**) and representative examples after culture with R848 and IL-2 (**B**) or IL-6 (**D**). Gates in **B**–**D** derived from CD3^−^CD19^+^ cells. Relative to CD19^+^ cells at Day 0, the CD27^++^CD38^++^ cells generated at Day 6 in culture showed upregulation of mRNA encoding BLIMP1 (*PRDM1*), *XBP1*, *IRF4* and downregulation of *BCL6* and *PAX5*. Data normalized to the housekeeping reference gene *RPLPO* (**E**).

Therefore, a cell culture model was developed to expand and differentiate B cells into ASCs *in vitro*. This model used a variety of conditions reported to generate ASCs from B cells (Ettinger *et al.*, 2005; Lanzavecchia and Corti, 2014), with a focus on conditions that: (i) mimicked T cell help (IL-2, IL-21 ± CD40L); (ii) simulated infections, which are hypothesized triggers of NMO relapses (IL-1β, TNFα and toll-like receptor stimulation with R848) (Koga *et al.*, 2011); and (iii) utilized cytokines already implicated in NMO (IL-6, BAFF, APRIL) (Kothur *et al.*, 2016). As shown in Fig. 2, after 6 days *in vitro*, select culture conditions were observed to promote the generation of ASCs (CD19^+^CD27^++^CD38^++^, Fig. 2A). This effect was particularly marked in conditions containing IL-2 and R848 (example in Fig. 2B and C) and, less so, in the presence of IL-21-predominant conditions with CD40L. As expected, this differentiation was minimal or absent in conditions known to maintain, but not proliferate, ASCs (BAFF plus IL-6, BAFF plus APRIL and IL-6 alone, example in Fig. 2C and D), and was equivalent to the condition without cytokines (Fig. 2A). The differentiated CD19^+^CD27^++^CD38^++^ ASCs upregulated the transcription factors *PRDM1*, *XBP1* and *IRF4*, downregulated *PAX5* and *BCL6* (Fig. 2E), and some expressed CD20 (mean 60%, range 18–93) and CD138 (mean 15%, range 1–41, Supplementary Fig. 2C and D). These observations verify the *in vitro* generation of early human ASCs.

### Antibody determination

**Figure 3:**
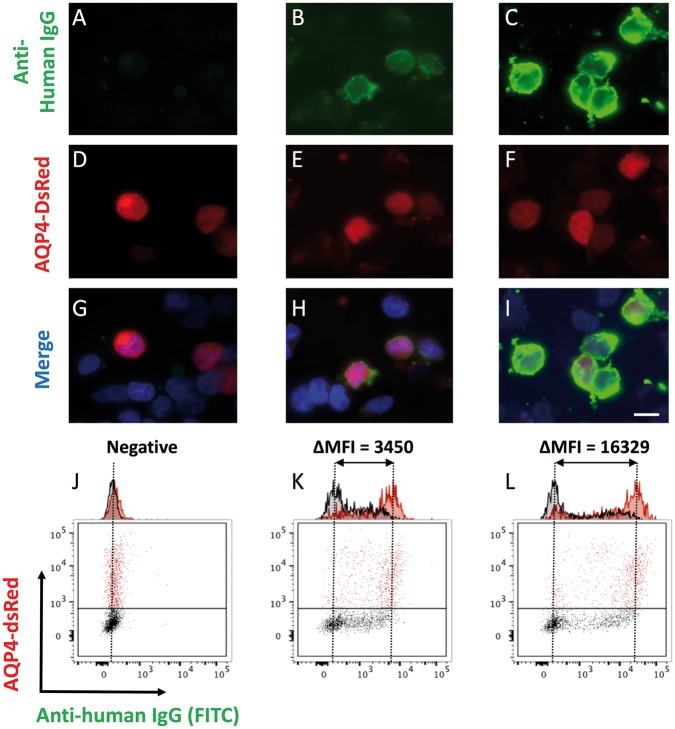
**Determination of AQP4-IgG from serum and culture supernatants.** Binding of patient IgG from culture supernatants (**A**–**C**; green, magnification ×1000; Scale bar = 10 µm) to live HEK-293T cells co-expressing AQP4 and dsRed from a single plasmid (**D**–**F**). Merge shown with DAPI (**G**–**I**). Sera or supernatants found to be positive by this cell-based assay, underwent flow cytometry to generate quantitative titres, defined as the difference in MFI between the dsRed-expressing (red) and dsRed-negative (black) cell populations (expressed as ΔMFI, **J**–**L**). Examples of negative (**A**, **D**, **G** and **J**), moderately (**B**, **E**, **H** and **K**) and strongly (**C**, **F**, **I** and **L**) positive samples shown.

**Figure 4:**
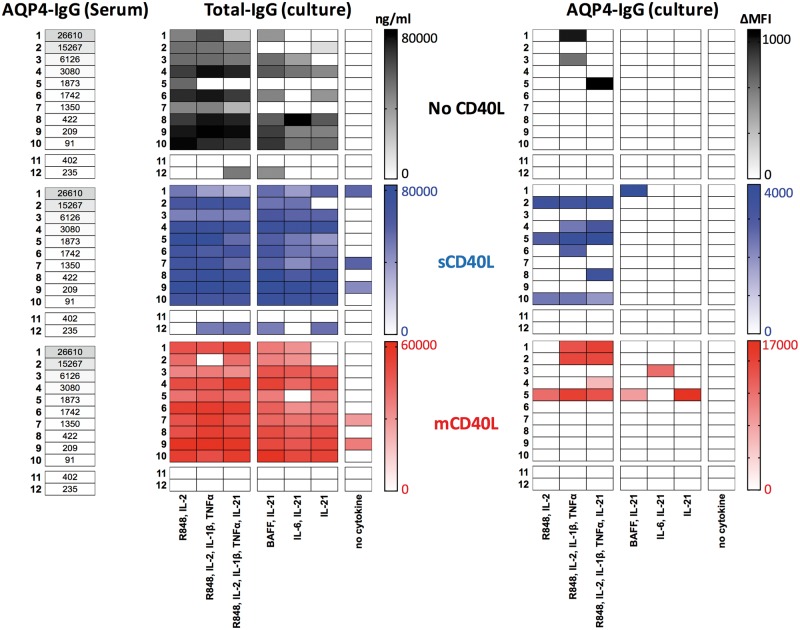
**Relationships between serum AQP4-IgG (ΔMFI), and the *in vitro* generation of total-IgG (ng/ml) and AQP4-specific IgG (ΔMFI) across culture conditions.** Heat-map generated from data on 12 patients (rows 1–12, corresponding to Table 1) across seven culture conditions. The first three culture conditions include R848 and IL-2 ± TNFα, IL-1β and IL-21, the next three include IL-21 ± BAFF or IL-6, and the seventh has no cytokines. Black = no CD40L; blue = with soluble CD40L (sCD40L); red = with membrane-bound CD40L (mCD40L). Grey bars with values in cells indicate serum AQP4-IgG levels (ΔMFI). Gamma-corrected values depicted, with absolute values shown in Supplementary Fig. 3. Patients are ordered by serum AQP4-IgG levels; Patients 11 and 12 received rituximab.

Next, after 13 days *in vitro*, total IgG levels and AQP4-IgGs were determined from all culture supernatants. Live CBA was used to determine the presence or absence of supernatant AQP4-IgGs binding exclusively to dsRed-expressing cells (examples in Fig. 3A–I). Subsequently, supernatants with visually-confirmed AQP4-IgGs were quantified by flow cytometry (Fig. 3J–L). The results are represented as heat maps in Fig. 4, with absolute values in Supplementary Fig. 3.

### *In vitro* generation of IgG

Without cytokines, there was limited IgG production from PBMC cultures despite CD40L. In the presence of R848 and IL-2 (±TNFα, IL-1β and IL-21) substantial quantities of IgG were generated in all patients, other than the two rituximab-treated patients, as expected (Patients 11 and 12, Fig. 4). IgG production was not enhanced by addition of CD40L (*P = *0.64, Mann-Whitney U-test, Supplementary Fig. 4A) and did not appear related to type or doses of immunotherapies, use of fresh or frozen PBMCs (Supplementary Fig. 2B) or relative circulating B cell subset frequencies (Supplementary Fig. 2E–G). In the three conditions containing IL-21 without IL-2, the addition of CD40L enhanced IgG production (*P = *0.0002, Mann-Whitney U-test, Supplementary Fig. 4B). Overall, the IgG levels per well correlated with the percentage of CD19^+^CD27^++^CD38^++^ ASCs generated per well (Spearman’s r = 0.71, *P < *0.0001) and a similar relationship was seen for tetanus IgG (Spearman’s r = 0.46, *P = *0.0084; Supplementary Fig. 4C).

### Correlations of *in vitro* generation of AQP4-IgG

**Figure 5:**
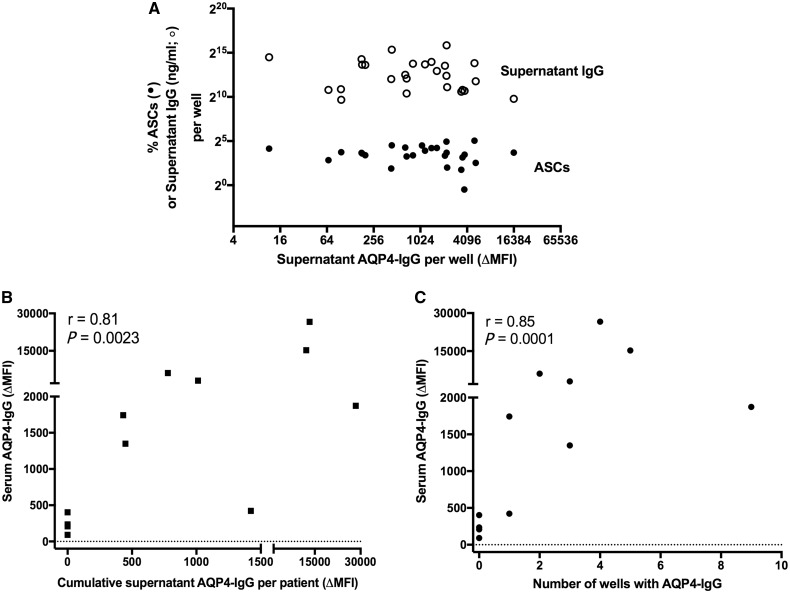
**Correlations with *in vitro* generated levels of AQP4-IgG.** The levels of AQP4-IgG in supernatants (**A**; ΔMFI) showed no correlation with the percentage of antibody secreting cells (ASCs, closed circles) per well, or the supernatant levels of total IgG (ng/ml, open circles), but showed a strong correlation with the serum AQP4-IgG (**B**; ΔMFI, Spearman’s r = 0.81, *P = *0.0023). Also, the number of culture wells with AQP4-IgG correlated well with serum AQP4-IgG levels (**C**; Spearman’s r = 0.85, *P = *0.0001).

However, across the wide range of supernatant AQP4-IgG levels generated *in vitro* (Fig. 4), the percentage of ASCs observed per well and the total IgG generated per well showed little variation (Fig. 5A and Supplementary Fig. 4C). AQP4-IgGs were almost exclusively detected in culture supernatants in the presence of CD40L (3/72 wells without CD40L versus 25/144 with CD40L, *P = *0.005, Fisher’s *t*-test, Fig. 4). Furthermore, AQP4-IgGs were detected in 24/108 wells with CD40L, R848 and IL-2, and this was often enhanced with addition of IL-1β, TNFα ± IL-21 (Fig. 4). Yet, only 4/108 wells containing IL-21 without IL-2, all with CD40L, showed AQP4-IgG production (*P < *0.0001, Fisher’s *t*-test). AQP4-IgGs were not detected in supernatants of PBMCs cultured under identical conditions from 12 age-matched healthy control subjects or the two rituximab-treated NMOSD patients.

Next, clinical factors that may impact on the generation of AQP4-IgGs *in vitro* were examined. Strikingly, the total supernatant AQP4-IgG production per patient and the number of AQP4-IgG containing wells per patient correlated well with their serum AQP4-IgG levels (Spearman’s r = 0.81, *P = *0.0023; Fig. 5B, and Spearman’s r = 0.85, *P* = 0.0001; Fig. 5C), and PBMCs from patients with high serum AQP4-IgG levels consistently showed *in vitro* AQP4-IgG production. In contrast, the total amount of AQP4-IgGs generated *in vitro* per patient did not correlate with the duration or number of immunotherapies, dose of corticosteroids or mycophenolate mofetil, time from illness onset or the time since the last clinical relapse (Supplementary Fig. 4D). Therefore, the serum AQP4-IgG level was the only identifiable parameter to correlate with the capacity of peripheral B cells to produce AQP4-IgGs *in vitro*.

### Naïve B cells express AQP4-specific immunoglobulins

**Figure 6:**
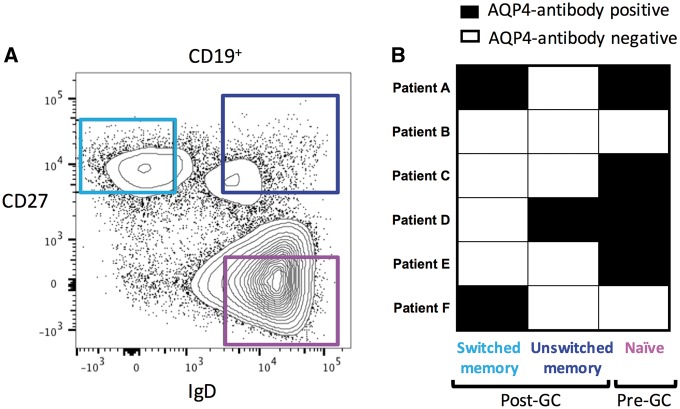
**Culture of *ex vivo* memory and naïve B cells in isolation**. Gating strategy to purify switched memory B cells (CD19^+^CD27^+^IgD^−^, light blue), unswitched memory B cells (CD19^+^CD27^+^IgD^+^, dark blue) and naïve B cells (CD19^+^CD27^−^IgD^+^, purple) from six patients with high levels of serum AQP4-IgGs (**A**; Patients A–F). After culture with mCD40L plus R848, IL-2, TNFα, IL-1β and IL-21) or with *ex vivo* activated autologous CD4 T cells, supernatants showed AQP4-IgG production (black boxes) from post-germinal centre (GC, three of six patients) and pre-germinal centre (four of six patients) B cells (**B**). IgG production in culture was measured for the three cell subsets (mean plus standard deviation): naïve = 654 ng/ml ± 280; unswitched memory = 1562 ng/ml ± 957; switched memory = 12 568 ng/ml ± 2098.

Finally, to explore which subset(s) of the peripheral B cells had the capacity to produce AQP4-IgGs, and gain insights into where B cell tolerance was disturbed in NMOSD, pre- or post-germinal centre B cell populations (gating strategy in Fig. 6A) were isolated *ex vivo* from six patients with high levels of serum AQP4-IgGs (ΔMFI > 2000). These B cell subsets were differentiated *in vitro* with either the combination of cytokines, which most robustly led to production of AQP4-IgGs in the above experiments (mCD40L plus R848, IL-2, TNFα, IL-1β and IL-21), or with *ex vivo* activated autologous CD4 T cells. The post-germinal centre class-switched (CD19^+^CD27^+^IgD^−^) and unswitched (CD19^+^CD27^+^IgD^+^) memory B cells produced AQP4-IgGs in three of six patients (Fig. 6B). However, surprisingly, naïve germinal centre-inexperienced B cells (CD19^+^CD27^−^IgD^+^) showed the capacity to produce AQP4-reactive IgGs in four of six patients (Fig. 6B), strongly implicating a pre-germinal centre defect in B cell tolerance led to the emergence of circulating AQP4-specific B cells in these patients.

## Discussion

This study established a multiparameter *in vitro* human culture system to characterize the contribution of peripheral B cells to the production of serum AQP4-IgGs, and generated several novel insights. First, circulating B cells differentiated *in vitro* and were able to produce AQP4-IgGs in the absence of antigen. The factors that induced AQP4-IgGs did so from unfractionated PBMC cultures at physiological concentrations without complex cytokine combinations. These minimalist culture requirements, which aimed to mimic known likely clinical associations of NMOSD such as concurrent infections (Koga *et al.*, 2011; Varrin-Doyer *et al.*, 2012), increase the likelihood that these or other similar factors operate *in vivo*, and show antigen is not necessary for ongoing AQP4-IgG production. Second, the AQP4-IgG-specific B cells were detected despite often intensive immunotherapy regimes and long intervals from last relapse. It may be that these cells are reactivated by immunotherapy withdrawal, which frequently leads to clinical relapses (Jarius *et al.*, 2008; Kim *et al.*, 2011). Indeed, their persistence may explain the frequent requirement for chronic immunosuppression in NMOSD patients. Third, the quantity of AQP4-IgGs produced *in vitro* by these B cells correlated well with the patient’s serum AQP4-IgG levels, suggesting they make an important—and perhaps sufficient—contribution to the circulating AQP4-IgGs. As the levels of total IgG and ASCs did not correlate with the production of AQP4-IgGs, it is likely that the baseline frequency of circulating AQP4-specific B cells reflected the serum AQP4-IgG level. Finally, these AQP4-IgGs were not derived from *ex vivo* circulating ASCs but were generated in culture from both antigen-experienced memory B cells and from naïve B cells, suggesting the intriguing possibility that a pre-germinal centre failure of B cell tolerance is an initiating factor in the generation of some AQP4-IgGs, and should be a population specifically targeted by immunotherapies. Our observations have important implications for understanding the mechanisms of AQP4-IgG generation and perpetuation, and provide a highly-translational model platform for the future study of lymphocyte drug-sensitivities and immune tolerance checkpoints in the field of antibody-mediated disorders.

The presence of circulating AQP4-specific CD20^+^ B cells and the earliest *in vitro*-derived CD20^+^ ASCs has several important clinical implications. It explains why anti-proliferative agents (including azathioprine and mycophenolate mofetil) and CD20-directed therapeutic monoclonal antibodies are efficacious in observational studies of NMOSD (Cree *et al.*, 2005; Jacob *et al.*, 2009; Costanzi *et al.*, 2011; Damato *et al.*, 2016). It also provides a rationale for the, occasionally precipitous, falls in AQP4-IgGs after rituximab (Kim *et al.*, 2011; Waters *et al.*, 2012; Valentino *et al.*, 2016). This is less well accounted for by the previous observation of CD20^−^ circulating plasmablasts as the cell type responsible for the production of AQP4-IgGs (Chihara *et al.*, 2011). However, more typically, rituximab has a modest effect in reduction of serum AQP4-IgG levels (Kim *et al.*, 2011; Pellkofer *et al.*, 2011). Yet, if AQP4-IgG is largely derived from ongoing differentiation of circulating B cells, deletion of CD20^+^ cells by rituximab should consistently reduce AQP4-IgG levels. Explanations for this apparent paradox include a role for CD20^−^ bone marrow plasma cells or that the non-depleted serum AQP4-IgGs post-rituximab may reflect the observed inability of rituximab to effectively deplete B cells within lymphoid organs (Kamburova *et al.*, 2013; Leandro, 2013), alongside the reactive post-rituximab B cell reconstitution from bone marrow-derived early-lineage autoreactive B cells (Kim *et al.*, 2011).

Indeed, our studies suggest that some of these bone marrow emigrant antigen-inexperienced naïve B cells have the potential to produce antibodies which target the extracellular domain of natively-expressed AQP4. This intriguing finding suggests that AQP4-specific B cells can be generated prior to the cognate T cell help and antigen exposure in germinal centres. This early loss of B cell tolerance may produce a pool of preformed AQP4-specific CD27^−^ naïve B cells, which are then activated by factors, including those in our culture system, to differentiate into ASCs and secrete AQP4-IgGs. The different factors known to activate naïve versus memory B cells may account for the modest variation in conditions that promote AQP4-IgG generation from a given patient’s unfractionated PBMCs (Deenick *et al.*, 2013). Alternatively, an explanation, which is more parsimonious with available literature, could invoke cognate germinal-centre AQP4-reactive T cells as a ‘second-hit’ in the development of memory B cells with high-affinity potentially pathogenic AQP4-IgGs (Varrin-Doyer *et al.*, 2012). This hypothesis is consistent with CD27^+^ B cell re-emergence as an accurate predictor of relapses (Kim *et al.*, 2011), our detection of AQP4-IgG derived from both CD27^+^ switched- and unswitched-memory B cells in culture, and the close clonal relationships recently observed between some CSF AQP4-specific plasma cells and peripheral CD27^−^ B cells (Kowarik *et al.*, 2017). To ensure these naïve cells do not represent stray memory B cells, in addition to isolated heavy chain sequencing (Kowarik *et al.*, 2015, 2017), future experiments should aim to reconstruct and contrast the cognate heavy and light chain pairs of these naïve and memory AQP4-reactive peripheral B cells, and relate their mutation load to CSF ASCs (Bennett *et al.*, 2009).

The peripheral AQP4-IgG producing ASCs generated *in vitro* from B cells must exist in patients but were not observed in circulation from our experiments. One possibility is that the AQP4-IgGs were present in ASC supernatants below the sensitivity limit of our assay, and may only be detected with high-throughput single-cell cloning approaches. It may also be that immunotherapies or often long durations since first relapse resulted in fewer circulating AQP4-specific ASCs. Another possibility is the generation of very short-lived plasma cells within lymph nodes, which die rapidly after secretion of AQP4-IgGs. Alternatively, cells may home to niches including lymph nodes, spleen or the CNS. Indeed, observed enrichment of AQP4-specific ASCs in the CSF of patients with NMOSD suggests this may be a preferential site of their sequestration (Bennett *et al.*, 2009), and the evolution of AQP4-reactive B cells from periphery to CNS, and their relationships to the secreted AQP4-IgGs, are the subject of important ongoing work (Kowarik *et al.*, 2015, 2017).

In a disease characterized by AQP4-IgG production over years, sometimes decades (Leite *et al.*, 2012), this model may present a deviation from traditional paradigms of antibody production that implicate the non-proliferative, CD20-negative bone marrow-niched long-lived plasma cells as major secretors of long-lived serum antibodies (Leite *et al.*, 2012; Stüve *et al.*, 2014; Alexander *et al.*, 2015; Bennett *et al.*, 2015; Halliley *et al.*, 2015). Without direct sampling from this compartment, our study does not fully exclude a contribution from bone marrow-niched long-lived plasma cells. Indeed, our data could represent a higher resting frequency of circulating AQP4-specific B cells in patients who also have more bone marrow resident AQP4-specific plasma cells. However, no patients with high serum AQP4-IgG levels showed low *in vitro* production of AQP4-IgGs, which would have been suggestive of a dominant plasma cell clone outside the circulation. Furthermore, the infrequent *in vitro* generation of AQP4-IgGs from patients with the lowest serum AQP4-IgG levels is likely to reflect the limited amplification of infrequent antigen-specific cells rather than a contribution from long-lived plasma cells. Indeed, PBMC cultures from all patients with the higher serum AQP4-IgG levels produced AQP4-IgGs *in vitro*.

The culture system we established presents other inherent experimental limitations. Because of widespread clinical recognition of NMOSD, all patients had received some immunotherapy prior to sampling, implying immunotherapy-responsive cell populations may have been deleted *in vivo*. However, treatment regimens were similar between our cohort and those from another study which reported an AQP4-IgG secreting ASC population (Chihara *et al.*, 2011). The major difference between these studies appears to be the ΔMFIs, which ranged between 67 and 16 329 (mean of 2458) in our data, versus a mean of ∼6 from Chihara *et al.* (2011). Also, in future longitudinal studies, the 30 culture conditions per patient could be expanded to better understand patient heterogeneity, as the current study was not exhaustive in varying these parameters. Nor did we explore the polyclonality or direct pathogenicity of *in vitro* secreted AQP4-IgGs, but it is likely that most IgGs reactive with the extracellular domain of AQP4 have pathogenic potential. Finally, the possibility of somatic hypermutations introduced in culture cannot be excluded, but *in vitro* somatic hypermutations are rare in the absence of antigen, especially without CD38 ligation (Bergthorsdottir *et al.*, 2001).

Nevertheless, our data largely reconcile clinical and therapeutic observations with the underlying B cell immunology from patients with NMOSD. The model of circulating B cell contributions to serum AQP4-IgGs may guide future therapeutic strategies and can be tested in prospective clinical treatment trials. Indeed, this *in vitro* model offers an opportunity to investigate tailored approaches within patients with antibody-mediated disorders where specific sets of conditions, which promote or inhibit pathogenic antibody production, may vary between individuals. This system may predict the influence of an individual’s cytokine milieu in their generation of pathogenic autoantibodies, and this knowledge could subsequently translate to blockade of an antibody-promoting pathway *in vivo*. Furthermore, this approach is successfully being translated to patients with other autoantibodies (S. R. Irani, unpublished results), and may prove to be an informative platform across autoantibody-mediated diseases.

## Supplementary Material

Supplementary Figures and Tables

## Abbreviations

AQP4: aquaporin-4
ASC: antibody-secreting cell
IgG: immunoglobulin G
MFI: median fluorescence intensity
NMOSD: neuromyelitis optica spectrum disorder
PBMC: peripheral blood mononuclear cell
