# Supplementary material for: Condition-dependent generation of aquaporin-4 antibodies from circulating B cells in neuromyelitis optica
Source: Brain. 2018 Feb 13;141(4):1063–74. doi: 10.1093/brain/awy010 (PMC5889028; doi:10.1093/brain/awy010)
Supplement: Supplementary Figures and Tables [file supplementary_data_awy010.pdf]

Supplementary Material

Supplementary Figure 1. Summary of the B cell lineage differentiation and associated cell-surface phenotypes. Bone marrow emigrant naïve antigen-inexperienced B cells encounter antigen and T cells in a germinal centre. Germinal centres are most commonly located in lymph nodes and spleen. The T cells express CD40L and secrete IL-2, IL-21 and TNFα, amongst other factors which help naïve B cells differentiate into $CD27^+$ unswitched (IgD^+) and switched (IgG^+) memory B cells. These then differentiate into antibody-secreting cells (below the dashed line: plasmablasts, short- and long-lived plasma cells) whose survival is supported by IL-6, BAFF and APRIL. Short-lived plasma cells may reside in tissues including bone marrow. Long-lived plasma cells typically niche in the bone marrow, but can reside in the CNS in states of inflammation. Antibodies in blue=IgG, red=IgD; yellow=IgM.

Supplementary Figure 2. Effects of immunotherapies and antibody-secreting cell phenotype. A. Resting B cell subsets from patients with and without mycophenolate mofetil (MMF) administration. B. No effects of freeze-thaw on IgG production from PBMC cultures under varying conditions for cells with membrane-bound CD40L co-cultures. Very similar results obtained in conditions with soluble CD40L and without CD40L (not shown). CD138 (C, blue) and CD20 (D, black) cells are shown within the $CD19^+CD27^{++}CD38^{++}$ antibody-secreting cells generated *in vitro*. A mean of 60% of the $CD19^+CD27^{++}CD38^{++}$ antibody secreting cells expressed CD20 (mean 60%, range 18– 93) and a mean of 15% expressed CD138 (range 1-41). E. Total IgG production across all tested conditions in patients stratified by MMF, corticosteroids and their doses (F), and the percentage of B cell subsets in blood (G).

Supplementary Figure 3. Relationships between *in vitro* generation of total IgG (ng/ml) and AQP4-specific IgG (ΔMFI) across 21 culture conditions. Absolute values in cells are accompanied by corresponding heat maps. Black = no CD40L; blue = soluble CD40L (sCD40L); red = membrane-bound CD40L (mCD40L).

Supplementary Figure 4. *In vitro* culture observations. From wells with R848 and IL-2, supernatant IgG levels did not vary with addition of CD40L (A). From wells with IL-21, without IL-2, addition of CD40L (membrane or soluble) generated more IgG *in vitro* ($p=0.0002$, Mann Whitney U test; B). Total IgG per well correlated strongly with the

percentage of ASCs per well (black, Spearman's r = 0.71, p<0.0001), and more modestly with tetanus-IgG (IU/ml; blue, Spearman's r = 0.46, p=0.0084), but not with AQP4-IgG generated per well (C; red, ΔMFI). AQP4-IgG levels in culture supernatants did not vary with days from illness onset, days since last clinical relapse and duration of immunotherapy (time parameters, left y-axis) or with corticosteroid dose, dose of mycophenolate mofetil (MMF) or number of immunotherapies (D; immunotherapy parameters, right y-axis).

Supplementary methods

Peripheral Blood Mononuclear Cells were isolated from whole blood using a Ficoll gradient (Ficoll-Paque, GE Healthcare). Phases were separated by centrifugation at 400 g for 30 min at room temperature and slow deceleration. A 3mL sterile pastette was used to isolate the buffy coat layer in a 50mL conical tube. The cells were washed with PBS/1%BSA twice (200g, 10 minutes, RT, medium acceleration and deceleration). The cells were counted using 0.04% Trypan Blue exclusion and frozen at 10– 20 × 10^6 per mL per cryovial in B cell medium (RPMI 1640 without phenol red, 5% FBS, 1% Penicillin/Streptomycin, 1% Glutamax, 0.1% beta-mercaptoethanol, 0.1% IgG-depleted transferrin (20 µg/ml)) with 40% Fetal Bovine Serum (FBS) and 10% DMSO chilled on ice. Cryovials containing cells were placed in a CoolCell® Cell Freezing Container (BioCision) and maintained at −80 °C for 24-72 hours before being transferred to liquid nitrogen tanks.

To thaw cells, the cryovials were removed from liquid nitrogen tanks into dry ice containers. Vials were thawed immediately with shaking in a lukewarm water bath. No more than two cryovials were thawed at the same time. The cell suspension was transferred dropwise to a 50mL conical tube containing 9 ml cold B cell medium per 1 ml of thawed cell suspension. The cells were washed twice with B cell medium (200g, 10 minutes) and counted. Viability was assessed using 0.04% Trypan Blue exclusion and ranged from 70–85% of the fresh cells). Cells were re-suspended in B cell medium at appropriate cell concentrations.

Supplementary Table 1. B cell subsets from 12 NMOSD patients and age- (± 5 years) and sex-matched healthy control (HC) subjects. HCs listed in order of matching to the patients 1-12. Percentages represent gating strategies from Figure 1.

Patient number or Healthy control	Age	Sex	CD19+	Plasmablasts	Switched Memory	Unswitched Memory	Naïve	Double negative
1	41	f	2.65	1.36	17.4	9.93	66.9	4.43
2	67	f	5.33	0.2	4.52	2.86	89.7	2.73
3	36	f	6.89	0.19	10.6	6.83	79.5	2.91
4	54	f	16.9	0.51	11.4	11.4	73	4.08
5	44	m	5.09	0.27	4.19	3.14	75.7	16.7
6	55	f	22.6	0	4.17	2.34	90.7	2.78
7	68	f	6.54	0.095	26	35.7	35.1	3.72
8	77	f	4.6	0.11	30.6	21.7	41.5	6.11
9	54	f	6.19	0.31	22.2	20.9	54.2	2.4
10	18	f	10.8	0.13	19.1	8.27	68	4.6
11	59	f	0.002	0	0	0	100	0
12	38	f	0.079	0	31.8	0	16.7	51.5
HC	36	f	6.93	0.28	20	26.7	68.8	3.11
HC	66	f	10.8	0.026	16.3	18.6	43.3	3.72
HC	34	f	8.76	0.081	24.9	28.2	49.5	3.9
HC	50	f	3.16	0.058	17	21.1	77.1	1.7
HC	43	m	7.04	0.3	4.96	16	57.2	4.69
HC	56	f	8.2	0.089	12.3	13.8	77.4	3.23
HC	72	f	7.75	0.25	10.8	4.25	72.3	1.69
HC	76	f	3.06	1.11	5.28	13.3	75	2.18
HC	50	f	5.86	1.76	6.45	11.2	61.9	3.2
HC	21	f	14.3	0.18	6.85	2.27	88.5	2.23
HC	61	f	7.51	0.19	13.4	9.33	82	2.84
HC	28	f	8.89	0.63	12.9	14.9	78.6	1.89

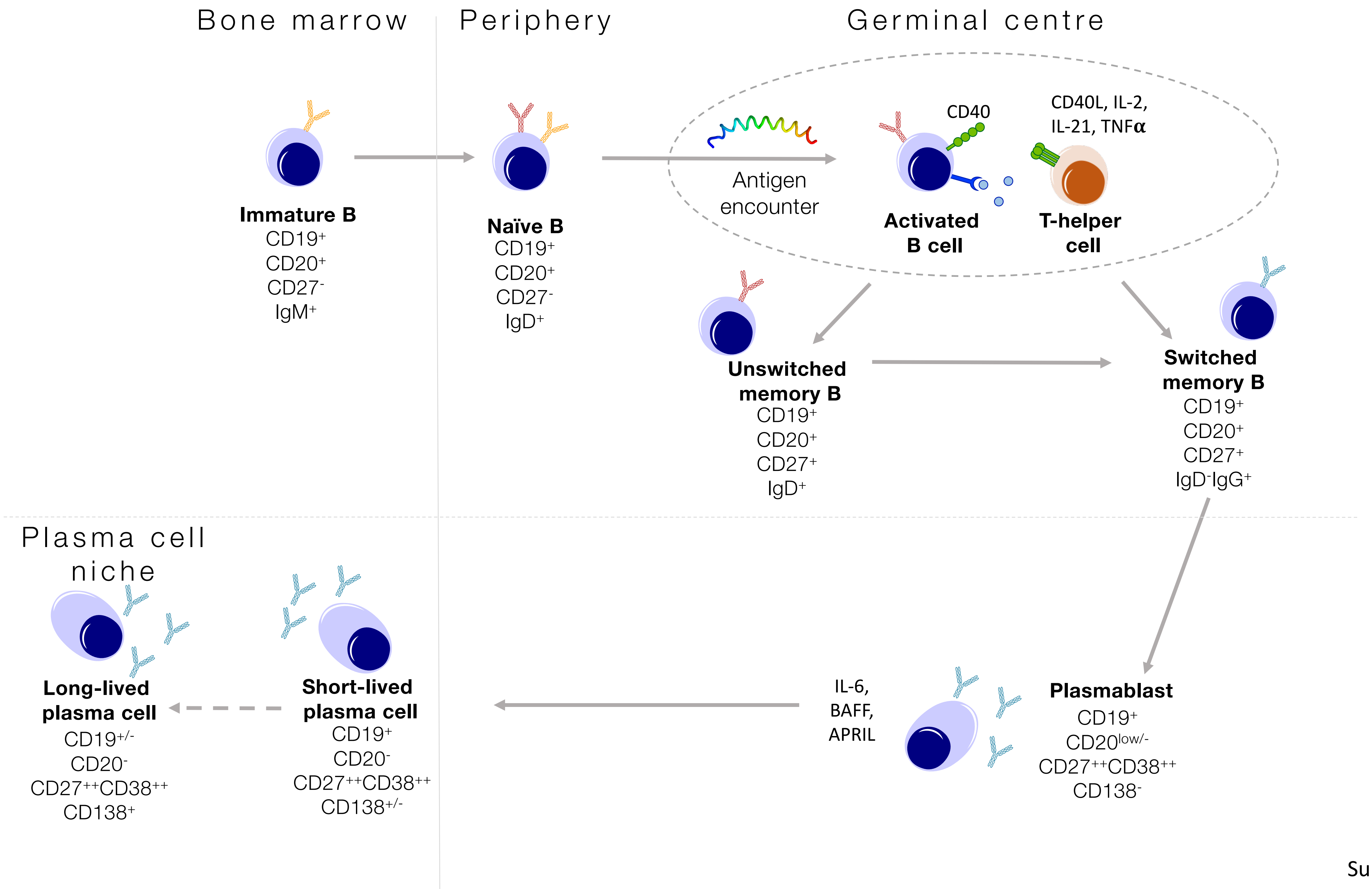

Supplementary Figure 1

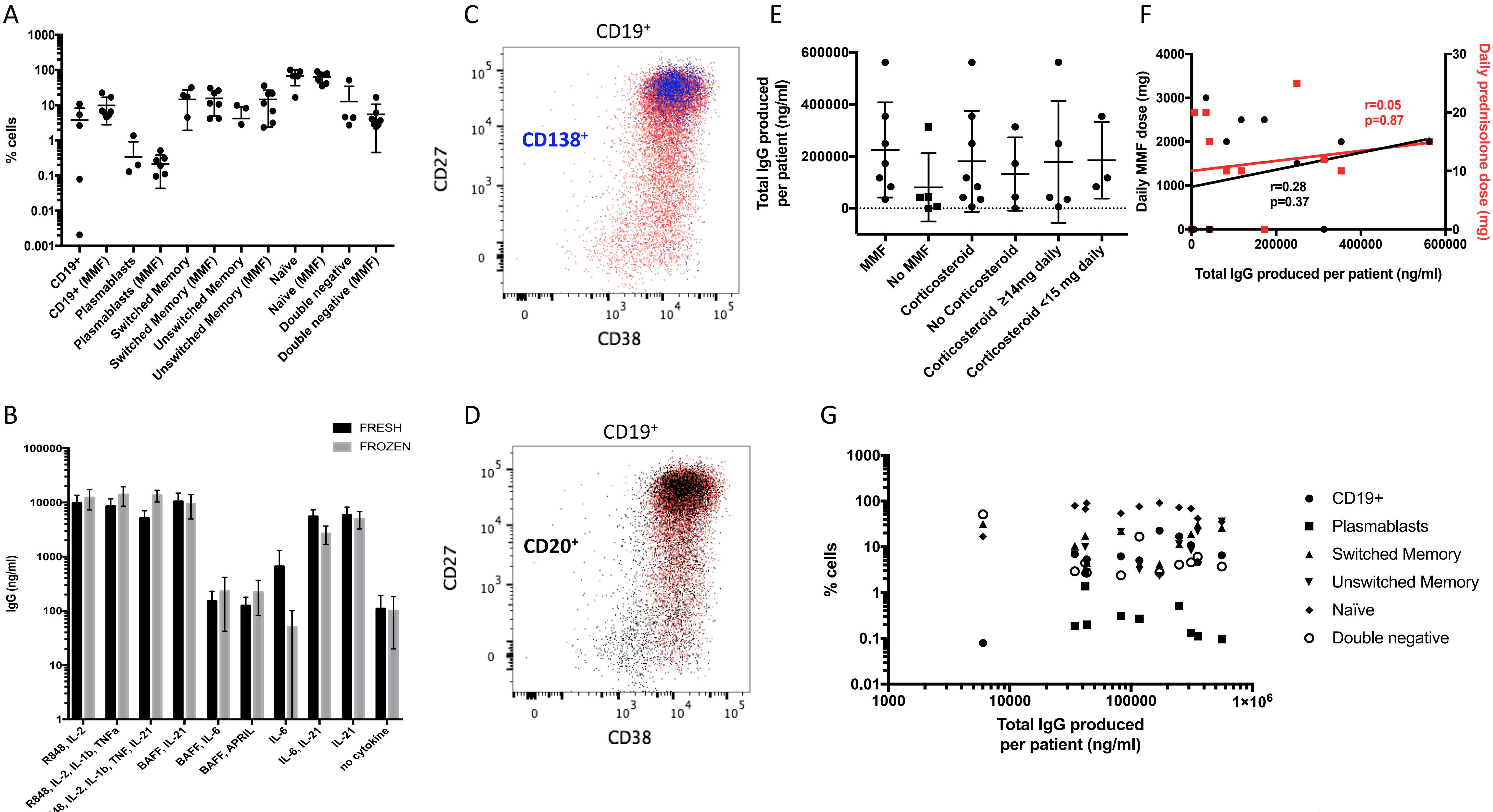

Supplementary Figure 2

Total-IgG (culture)

ng/ml

No CD40L

Patients	R848, IL-2	R848, IL-2, IL-1β, TNFα	R848, IL-2, IL-1β, TNFα, IL-21	BAFF, IL-21	IL-6, IL-21	IL-21	no cytokine
1	734	5795	40	312	0	0	0
2	1557	1291	704	0	0	27	0
3	1742	1874	989	1705	200	0	0
4	10912	42452	24611	3889	1233	531	0
5	2072	0	0	0	0	0	0
6	20212	38008	12354	699	0	306	0
7	630	727	79	0	0	0	0
8	12082	38835	25100	3244	79129	3765	0
9	37861	59783	44387	10483	746	762	0
10	48150	20472	14253	14448	1153	1566	0
11	0	0	0	0	0	0	0
12	0	0	947	414	0	0	0

Scale: 0, 20000, 40000, 60000

sCD40L

Patients	R848, IL-2	R848, IL-2, IL-1β, TNFα	R848, IL-2, IL-1β, TNFα, IL-21	BAFF, IL-21	IL-6, IL-21	IL-21	no cytokine
1	958	170	85	1643	193	2510	2187
2	7871	13185	11767	1184	1258	0	0
3	667	767	676	6386	2366	2360	0
4	17912	19878	13863	19319	14062	16060	0
5	41849	58625	1772	3335	859	218	0
6	12307	4148	8493	7820	1089	633	0
7	12899	12660	1766	2694	390	1899	2540
8	14443	30713	16030	43373	4886	22694	0
9	42122	51569	30403	43624	40394	23617	437
10	10838	9266	8596	26686	16335	18534	0
11	0	0	0	0	0	0	0
12	0	856	1290	743	0	1417	0

Scale: 0, 20000, 40000, 60000, 80000

mCD40L

Patients	R848, IL-2	R848, IL-2, IL-1β, TNFα	R848, IL-2, IL-1β, TNFα, IL-21	BAFF, IL-21	IL-6, IL-21	IL-21	no cytokine
1	6236	5391	14514	878	301	0	0
2	1747	0	1538	635	331	0	0
3	561	887	384	5706	4325	2438	0
4	8268	6167	23063	15100	2188	9048	0
5	1348	3494	2198	816	0	882	0
6	19640	21426	18656	2926	356	969	0
7	6079	17870	7672	2265	1223	1830	203
8	7110	19199	13336	8509	3092	7372	0
9	53187	58397	31865	10121	4787	12360	814
10	19116	7469	22258	47908	10145	15321	0
11	0	0	0	0	0	0	0
12	0	0	0	0	0	0	0

Scale: 0, 20000, 40000

AQP4-IgG (culture)

ΔMFI

No CD40L

Patients	R848, IL-2	R848, IL-2, IL-1β, TNFα, IL-21	R848, IL-2, IL-1β, TNFα, IL-21	BAFF, IL-21	IL-6, IL-21	IL-21	no cytokine
1	0	653	0	0	0	0	0
2	0	0	0	0	0	0	0
3	0	97	0	0	0	0	0
4	0	0	0	0	0	0	0
5	0	0	1074	0	0	0	0
6	0	0	0	0	0	0	0
7	0	0	0	0	0	0	0
8	0	0	0	0	0	0	0
9	0	0	0	0	0	0	0
10	0	0	0	0	0	0	0
11	0	0	0	0	0	0	0
12	0	0	0	0	0	0	0

Scale: 0, 500, 1000

sCD40L

Patients	R848, IL-2	R848, IL-2, IL-1β, TNFα, IL-21	R848, IL-2, IL-1β, TNFα, IL-21	BAFF, IL-21	IL-6, IL-21	IL-21	no cytokine
1	0	0	0	3808	0	0	0
2	1669	1178	2124	0	0	0	0
3	0	0	0	0	0	0	0
4	0	179	822	0	0	0	0
5	439	2233	3571	0	0	0	0
6	0	432	0	0	0	0	0
7	0	0	0	0	0	0	0
8	0	0	1423	0	0	0	0
9	0	0	0	0	0	0	0
10	182	200	67	0	0	0	0
11	0	0	0	0	0	0	0
12	0	0	0	0	0	0	0

Scale: 0, 1000, 2000, 3000

mCD40L

Patients	R848, IL-2	R848, IL-2, IL-1β, TNFα, IL-21	R848, IL-2, IL-1β, TNFα, IL-21	BAFF, IL-21	IL-6, IL-21	IL-21	no cytokine
1	0	2223	5101	0	0	0	0
2	0	3772	3450	0	0	0	0
3	0	0	0	0	682	0	0
4	0	0	12	0	0	0	0
5	682	5292	2268	98	0	16329	0
6	0	0	0	0	0	0	0
7	0	0	0	0	0	0	0
8	0	0	0	0	0	0	0
9	0	0	0	0	0	0	0
10	0	0	0	0	0	0	0
11	0	0	0	0	0	0	0
12	0	0	0	0	0	0	0

Scale: 0, 5000, 10000, 15000

Supplementary Figure 3

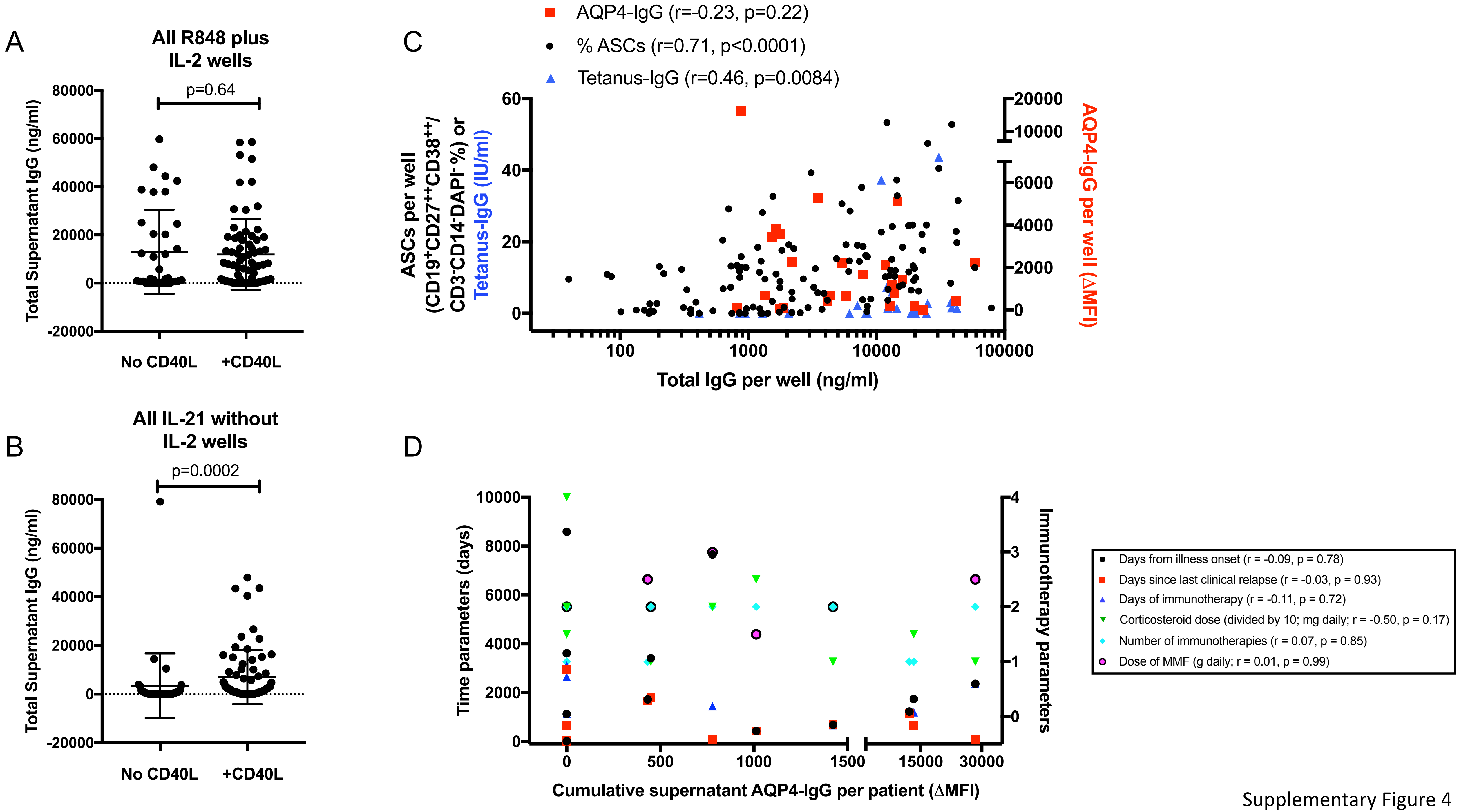

Supplementary Figure 4
